# Managing Care during the COVID-19 Pandemic: The Point of View and Fears of Pediatric Cancer Patients’ Families

**DOI:** 10.3390/children9040554

**Published:** 2022-04-13

**Authors:** Olga Nigro, Giovanna Sironi, Andrea Ferrari, Gabriele Tinè, Gabriele Infante, Francesco Barretta, Matteo Silva, Carlo Alfredo Clerici, Stefano Chiaravalli, Elisabetta Schiavello, Veronica Biassoni, Marta Podda, Cristina Meazza, Filippo Spreafico, Michela Casanova, Monica Terenziani, Roberto Luksch, Maura Massimino

**Affiliations:** 1 Pediatric Oncology Unit, Fondazione IRCCS Istituto Nazionale dei Tumori, 20133 Milan, Italy; olga.nigro@istitutotumori.mi.it (O.N.); giovanna.sironi@istitutotumori.mi.it (G.S.); andrea.ferrari@istitutotumori.mi.it (A.F.); matteo.silva@istitutotumori.mi.it (M.S.); stefano.chiaravalli@istitutotumori.mi.it (S.C.); elisabetta.schiavello@istitutotumori.mi.it (E.S.); veronica.biassoni@istitutotumori.mi.it (V.B.); marta.podda@istitutotumori.mi.it (M.P.); cristina.meazza@istitutotumori.mi.it (C.M.); filippo.spreafico@istitutotumori.mi.it (F.S.); michela.casanova@istitutotumori.mi.it (M.C.); monica.terenziani@istitutotumori.mi.it (M.T.); roberto.luksch@istitutotumori.mi.it (R.L.); 2 Clinical Epidemiology and Trial Organization Unit, Fondazione IRCCS Istituto Nazionale dei Tumori, 20133 Milan, Italy; g.tine@campus.unimib.it (G.T.); gabriele.infante@istitutotumori.mi.it (G.I.); francesco.barretta@istitutotumori.mi.it (F.B.); 3 Department of Oncology and Hemato-Oncology, University of Milan, 20100 Milan, Italy; carloalfredo.clerici@istitutotumori.mi.it; 4 SSD Clinical Psychology, Fondazione IRCCS Istituto Nazionale dei Tumori, 20133 Milan, Italy

**Keywords:** COVID-19, pediatric oncology

## Abstract

(1) Background: When the COVID-19 pandemic arrived, changes had to be made to several management aspects at our Pediatric Oncology Unit. We investigated how the families perceived these changes. (2) Methods: Two questionnaires were developed at the Pediatric Oncology Unit of the Istituto Nazionale dei Tumori in Milan in order to explore how the pandemic had affected the experience of patients who had been or were being treated at our hospital, as well as their families. These questionnaires were administered to three groups of individuals. (3) Results: Between July and October 2020, 120 questionnaires were administered to parents of patients. The impact of school closures and the impossibility of attending sports and social activities outside the hospital were regarded as important, and it was reported that 77.5% of parents judged social distancing to have affected their children. Regarding the changes introduced in the management of the ward and outpatient clinic, most parents’ opinions were positive. Differences in the opinions expressed by Groups 2 and 3 were statistically significant on the topics of relationships in the ward and staff workload. The aspect most negatively affected by the pandemic was the support that patients’ parents were able to give each other. Regardless of whether patients were treated before the pandemic or after the first lockdown, all parents indicated strong degrees of satisfaction with the care received and the organizational arrangements. (4) Conclusions: The results of our study point us in the right direction to further improve our daily work and better respond to the needs of our patients and their families.

## 1. Introduction

The COVID-19 pandemic greatly impacted all healthcare settings, obliging the healthcare community to try and deal with this unexpected emergency with profound effects on how patients and their families are managed.

Cancer units all over the world have found themselves striving to minimize the risk of the coronavirus spreading within hospitals while providing the best possible management of cases testing positive COVID-19 and continuing to guarantee the oncological care patients need [1,2,3,4,5].

When the pandemic arrived, drastic changes had to be made to several management aspects at our Pediatric Oncology Unit. Among other measures, access to the ward and outpatient clinic was restricted, even for educators, teachers, and volunteers (partly to comply with central government legislation). Even the physical presence of clinical psychologists on the wards had to be limited in many cases. Only one parent was allowed during a child’s hospital stay, and non-urgent visits were handled by telephone.

Patients and their families had to talk to doctors with masks over their faces or via impersonal phone calls, which made communications more challenging and added to the anguish of coping with cancer and all the “classical” issues that typically accompany a cancer diagnosis. The need for psychological support in such situations has been globally recognized, and most families were often under huge stress, but restrictions imposed to contain the pandemic and enforce social distancing measures left our patients and their families with fewer relational resources. They could no longer rely on the support of grandparents and friends. They had fewer chances to socialize through schooling activities. Group activities for psychosocial support were suspended. Only one parent could be involved in hospital stays [3,5].

Given this situation, we wished to better understand how the new care management arrangements were perceived by our healthcare service users. In particular, we wanted to explore how the direct and indirect consequences of the pandemic had affected the experience of patients who had been or were being treated at our hospital, as well as their families. A further goal of the study was to use questionnaires to determine whether any of the recent organizational adjustments had enhanced the parents’ experience in consideration of the possibility of adopting or retaining these changes in the future.

Another survey was previously conducted among our adolescent patients to assess their perception of the risk posed by the new coronavirus and their level of stress regarding the COVID-19 pandemic [6]. In the present study, we looked at the patients’ families, investigating how changes made to the way we work and approach patients and families since the arrival of the pandemic had been perceived.

## 2. Materials and Methods

Our Pediatric Oncology Unit has a very large patient pool of approximately 250 patients per year. Our patients are all affected by solid tumors; the majority of these are represented by tumors of the central nervous system and sarcomas.

As in the rest of Italy and the world, some of our patients have been infected with SARS-CoV-2.

Two questionnaires were developed by physicians and psychologists at the Pediatric Oncology Unit of the Istituto Nazionale dei Tumori in Milan in order to explore how the direct and indirect consequences of the pandemic had affected the experience of patients who had been or were being treated at our hospital, as well as their families. A further goal of the study was to use questionnaires to ascertain whether any of the recent organizational adjustments had enhanced the parents’ experience, with a view toward adopting or retaining these changes in the future.

The two separate questionnaires were administered to three different groups of individuals.

One questionnaire was administered to 40 parents (or caregivers) looking after 40 patients who experienced the organizational changes made to cope with the pandemic, i.e., patients who had begun their cancer treatments before the pandemic and continued to be treated during the outbreak (Group 1). This questionnaire was aimed to investigate concerns about the risk related directly to COVID-19 infection, the impact of social distancing measures on various aspects of the care pathways and family relationships, and the effects of management changes on the pediatric oncology ward and at the outpatient clinic compared to their previous hospital experience.

Another questionnaire was administered to 40 parents looking after 40 patients whose treatments stopped before the pandemic broke out (Group 2) and to another 40 parents looking after 40 patients whose treatments began after the arrival of the pandemic (Group 3). This second questionnaire was aimed to compare these two groups of parents (patients treated before versus after the outbreak of the COVID-19 pandemic), investigating their experience with issues such as the care they received, organizational aspects on the ward and at the outpatient clinic, and the social support network.

Parents were invited to complete the questionnaire during clinical visits or hospital stays. A staff member personally provided each parent a printed copy of the questionnaire and informed them about the aims of the study. The questionnaire was administered in an anonymous format and completed by parents in their own time without any assistance or supervision on the part of staff members, ensuring the confidentiality of all the collected data.

The questionnaire was approved by the Research Ethics Committee at the Fondazione IRCCS Istituto Nazionale dei Tumori, Milan, Italy (code 178/20 of the Italian National Observatory on Clinical Trials). All involved parents gave their written informed consent to their participation in the study.

The sample size of the study was not based on any specific objectives. The survey was purely descriptive and aimed to explore how the parents of patients in the care of the INT Pediatric Oncology Unit perceived the direct and indirect consequences of the COVID-19 pandemic.

The following demographic variables were collected for both questionnaires: parents’ age, gender, educational qualifications, region of origin, and occupation [7], together with the type of disease and age of the respective patients.

The first and second questionnaires consisted of 32 and 18 questions, respectively, with 5 possible answers: “Not at all”, “Slightly”, “Moderately”, “Very much” and “Extremely”.

The items in the first survey were grouped into six subscales concerning “perception and concerns about the risk associated with COVID-19 infection” (q1.01–q1.05); “impact of social distancing and lockdown measures on personal and family life” (q1.06–q1.13); “effects of changes in the management of the pediatric cancer ward and outpatient clinic compared with the situation before the pandemic” (q1.14–q1.18); “hospital experience relating to cancer treatments during the pandemic” (q1.19–q1.26); “perceptions relating to care providers during the pandemic” (q1.27–q1.30); and “suggestions for improving the support available” (q1.31–q1.32).

Parents’ and patients’ characteristics were summarized in terms of frequencies (absolute and relative) for qualitative variables and in terms of medians, first quartiles, and third quartiles for continuous variables.

For the first questionnaire, a descriptive analysis was conducted by grouping the items in the six above-mentioned subscales, obtaining a score for each by averaging the cumulative scores of the single items in a given subscale. For subscales 1, 2, 3, and 4, the lower the score, the lower the perception of the risk to parents and patients caused by the pandemic and the organizational changes made to cope with it. For subscales 5 and 6, the lower the score, the more positive the parents’ opinion of their experience. The following parameters were calculated for each subscale: mean, standard deviation (SD), median, first and third quartiles, and range of variation.

For the second questionnaire, the answers given were considered as categorical, continuous, and ordinal categorical, and a Fisher’s exact test [8] (F), a Wilcoxon–Mann–Whitney test [9] (WMW), and a Proportional Odds Model [10] (POM) were used, respectively, to assess differences between the answers given by Groups 2 and 3. The models were used to examine the adjusted differences in two steps. First we ran a POM for each item, including the parents’ age, gender, educational qualifications, and region of origin, as well as the patients’ age. Then, the values predicted with this model were extracted and input as covariates in a new POM to obtain the adjusted comparisons by means of Wald tests of the regression coefficient of the study group covariate. Absolute and relative frequencies were then calculated for each item. The analyses were run using R (version 4.0.3).

## 3. Results

Between July and October 2020, 120 questionnaires (40 for each study group) were administered to parents of patients at our Pediatric Oncology Unit (87/120 were administered between July and September and 33/120 were administered between September and October). Supplementary Table S1 shows the details of the parents (age, gender, educational qualifications, region of origin, and occupation) and patients (age and type of tumor) that were collected. The table shows that our questionnaires were mainly answered by patients’ mothers, who accounted for 60% of Group 1, 72.5% of Group 2, and 82.5% of Group 3. The mean ages of the sample were 47.5 years for Group 1, 50.5 years for Group 2, and 43.5 years for Group 3.

### 3.1. First Questionnaire

Table S2 shows the 32 questions and answers, while Table 1 shows the results grouped into subscales with the respective mean and median values.

We can see from these mean and median values in Table 1 that parents were concerned about COVID-19 infection, the impact of social distancing, and the effects of changes to care management and the hospital experience (subscales 1–4). On the other hand, they did not perceive a lack of healthcare staff and they approved of the improvements to care provision (subscales 5–6).

Analyzing Table S2 in detail shows that the impact of school closures was regarded as important: 52.5% of respondents felt that their children were from moderately (30%) to extremely (very much: 12.5%; extremely: 10%) affected by being unable to attend school (question 1.09). The impossibility of attending sports and social activities outside the hospital was also regarded as moderately (45.0%) to extremely (very much: 12.5%; extremely: 5.0%) important by 62.5% of parents (question 1.11). The impact of social distancing on daily life was mainly attributed to distancing from family members and friends (question 1.13): 77.5% of parents judged this to have affected their children from moderately (42.5%) to extremely (very much: 12.5%; extremely: 22.5%). Less than half of the parents answering the questionnaire (42.5%) reported experiencing economic difficulties or concerns compared to before the pandemic (question 1.07).

Regarding the changes introduced in the management of the ward and outpatient clinic (triage, strict time slots for medical visits, separate routes for patients according to need, “telephone consultations”, and single rooms), most parents’ opinions were positive (subscale 3 in Table 1, which shows a mean of 2.1, a median of 2.0, and a range of 1.8–2.4). There was also general satisfaction with the handling of emergencies and the support given to patients and families, with 85% of parents giving positive opinions (moderately: 15.0%; slightly: 57.5%; not at all: 27.5%) on these matters (question 1.14).

Meanwhile, 60% of parents found that restricting hospital access to only one of the parents was moderately (17.5% both for questions 1.19 and 1.20) to extremely (very much: 15.0% for question 1.19 and 25.0% for question 1.20; extremely: 27.5% for question 1.19 and 20.0% for question 1.20) difficult to cope with for both the family as a whole and the child receiving treatment (questions 1.19 and 1.20). The banning of visitors (family and friends) during hospital stays also had an impact according to 52.5% of respondents.

### 3.2. Second Questionnaire

Table 2 shows the results for each pair of items with the answers given by Groups 2 and 3, as well as the differences between them. Details of the *p*-values calculated with Fisher’s exact test and the Wilcoxon–Mann–Whitney test are provided in Supplementary Table S3.

Differences in the opinions expressed by Groups 2 and 3 were statistically significant on the topics of relationships on the ward and staff workload. Our findings suggest that the aspect most negatively affected by the pandemic was the support that patients’ parents were able to give each other, as seen from parents’ answers to question 2.11 (from 70.0% to 37.5% for “very much/extremely” responses and from 2.5% to 37.5% for “not at all/slightly” responses; *p* < 0.001).

Staff workload was perceived as moderately to extremely excessive by 95% of parents in Group 2 and 92.5% of those in Group 3, with internal shifts from “very much” to “moderately” from 47.5% to 17.5% and from 40.0% to 72.5%, respectively (*p* = 0.010 for question 2.14).

Regardless of whether patients were treated before the pandemic or after the first lockdown, all parents indicated a strong degree of satisfaction with the care received (more than 90% of moderately/very much/extremely responses to questions 2.01, 2.02, and 2.05) and the organizational arrangements (90% of moderately/very much/extremely responses to questions 2.12 and 2.13; 100% of moderately/very much/extremely responses to questions 2.03 and 2.04).

The financial impact of the disease and related treatments was considered less important (75% or less of “not at all/slightly” responses to q16). Family relations suffered slightly (from 67.5% to 47.5% and from 12.5% to 30.0% in “very much/extremely” and “not at all/slightly” responses, respectively) from the COVID-19 outbreak, and families’ social and emotional support remained an important factor (80% of “very much/extremely/moderately” responses to q18).

## 4. Discussion

Our study was aimed to investigate how the COVID-19 pandemic and consequent organizational adjustments at our hospital affected the experience of families already having to cope with their children’s cancer. As previously reported by other authors, the impact that this virus may have on the pediatric population and the management of children with cancer is unclear and poorly documented [11,12,13].

Our questionnaires revealed families’ fears concerning COVID-19, relating to the risk of contagion and the psychological repercussions of social distancing. Though parents’ answers indicated a moderate fear of the infection, they seemed more concerned about the impact of the pandemic on family relations and the habits of daily life.

All the collateral effects of the pandemic, as well as the changes to social habits imposed by the lockdown to contain it, were found to have had relevant impacts on the lives of patients and their families (in the economic sphere as well as). A crucial point underscored by our results concerns how social distancing translated into a limitation of family ties, isolating grandparents and thereby reducing families’ social and support networks.

Generally speaking, parents were very satisfied with the new steps taken to manage the ward and outpatient clinic (triage, strict time slots, separate routes for different patients’ needs, and single rooms). This shows that the healthcare operators managed to provide the necessary support for patients and families even in the complex times of the ongoing pandemic. None of our study groups suggested changes to how their children’s care was being managed. They were more critical of the restrictions imposed on access to the ward, with more than 60% of parents emphasizing the strong impact of the ban on the other parent, other family members, or friends visiting their child in hospital.

Our survey also highlighted the importance of relations between members of a patient’s family. The pandemic and related social distancing measures strongly reduced support among the involved families. Parents also lacked the help of people who shared the same experience and had to learn to spend more time in their ward rooms in forced isolation. Adolescent patients likewise missed the support that other patients on the ward could normally offer. As we have already reported in other papers, programs dedicated to teenagers (e.g., the Youth Project) also had to be converted into weekly videoconferences to comply with social distancing requirements [14,15].

Another area negatively affected by the pandemic concerns patients’ education. It is hard for schools to strike the right balance between establishing a set of rules to ensure the safety of teachers and other staff, complying with clinical precautions, and somehow resuming activities that are fundamentally important to our patients, as well as children and young people in general. Uncertainty about their prognosis and the need to concentrate on the treatment of their disease can lead to the delay of any plans for the future of a child diagnosed with cancer. Continuing their education in the classroom or in hospital can help to combat this harmful tendency [16]. Parents reported being from moderately to extremely concerned about their children’s schooling in 52.5% of cases. Due to the restrictions imposed nationwide by law during the first lockdown, our teachers had to continue the in-hospital school program via remote classrooms. However, the results of experience suggested that distance learning cannot meet the needs of particular students such as children and adolescents with cancer. Attending school in person plays a crucial part in motivating these patients to invest in their studies and future [16,17].

The psychology of children is very complex and difficult to comprehend. Additionally, in the “normal” pre-pandemic period, it was often difficult for children in treatment to live while maintaining a certain level of “normality”. School is a fundamental setting for not only education but also sociality and normality. Our child patients have sometimes found it difficult to cope with life outside the shell created in the hospital because they are no longer treated as “special” outside. Life outside the hospital is also made up of challenges, including the act of simply going to school, that must be address to ensure complete psychological support during the course of treatment. 

A further crucial aspect concerns the provision of families with adequate psychological support. During the first lockdown in the spring of 2020, legal restrictions obliged us to use modern telecommunications to try and provide this support. We were well-aware that, at best, this could only be “better than nothing” and far from ideal, so we did our best to restore more adequate in-person psychological support for patients and their families as soon as possible [5].

Our study had various limitations, including the facts that it was purely descriptive and the sample was limited in size and consisted largely of mothers. Our questionnaires had not been validated, and our results only describe the situation at our own institution, the habits and care methods adopted at our unit, and the changes we made to cope with the pandemic.

Another notable limitation concerns G1 respondents, who were from families whose children managed to cope with COVID-19 and continued their medical treatment. As consequence, G1 respondents obviously represented a bias. Our goal, however, was to understand the differences in our work from their points of view during the pre-pandemic and post-pandemic periods.

Despite these limitations, our study could promote discussion and raise awareness regarding a relevant aspect of young patients’ care. We believe that listening to parents is crucial to understanding how they have coped with these challenging times, as well as how changes to how their children’s care is managed have affected the experience of the disease pathway. Parent responses are fundamental now that our approach has transitioned from an “emergency management for a few weeks” to a “risk management for an unspecified period of time”. Though we cannot generalize our results, our patients’ and their parents’ opinions should be taken into consideration and help orient our approach to care.

The results of our study point us in the right direction in order to further improve our daily work and better respond to the needs of our patients and their families.

## Figures and Tables

**Table 1:** Six subscales of questionnaire 1.

Subscales		Mean (SD)	Median (First-Third Quartile)	Min–Max
**1**	**Concern about COVID-19 infection**	3.1 (0.9)	3.0 (2.7–3.4)	1.0–5.0
**2**	**Impact of social distancing**	2.6 (0.7)	2.5 (2.2–3.0)	1.2–4.9
**3**	**Effects of changes to care management compared with organization before the pandemic**	2.1 (0.5)	2.0 (1.8–2.4)	1.0–3.4
**4**	**Hospital experience related to cancer care during the pandemic**	2.7 (0.9)	2.6 (2.1–3.4)	1.1–4.5
**5**	**How parents perceived care provided by staff during the pandemic**	1.6 (0.6)	1.5 (1.0–1.8)	1.0–3.5
**6**	**Opinions regarding improvements to care provision**	1.3 (0.6)	1.0 (1.0–1.5)	1.0–3.5

**Table 2:** Questionnaire 2: results for each pair of items (Groups 2 and 3).

Item		Group	Not at All *n* (%)	Slightly *n* (%)	Moderately/Fairly *n* (%)	Very Much *n* (%)	Extremely *n* (%)	*p*-Value POM
**q2.01**	**Do you consider the medical care to have been clinically satisfactory?**	2	0 (0)	0 (0)	0 (0)	10 (25.0)	30 (75.0)	0.271
		3	0 (0)	0 (0)	1 (2.5)	15 (37.5)	24 (60.0)	
**q2.02**	**Do you think the medical care was satisfactory in terms of communication?**	2	0 (0)	1 (2.5)	2 (5.0)	15 (37.5)	22 (55.0)	0.311
		3	0 (0)	0 (0)	3 (7.5)	21 (52.5)	16 (40.0)	
**q2.03**	**Do you think the time spent by doctors during outpatient visits was adequate?**	2	0 (0)	0 (0)	6 (15.0)	13 (32.5)	21 (52.5)	0.756
		3	0 (0)	0 (0)	4 (10.0)	18 (45.0)	18 (45.0)	
**q2.04**	**Do you think the time spent by doctors during inpatient visits was adequate?**	2	0 (0)	0 (0)	6 (15.0)	14 (35.0)	20 (50.0)	0.936
		3	0 (0)	0 (0)	6 (15.0)	17 (42.5)	17 (42.5)	
**q2.05**	**Do you think the nursing care was satisfactory?**	2	0 (0)	0 (0)	2 (5.0)	16 (40.0)	22 (55.0)	0.101
		3	0 (0)	0 (0)	1 (2.5)	11 (27.5)	28 (70.0)	
**q2.06**	**Do you feel you received the psychological support you needed during your child’s treatment?**	2	0 (0)	4 (10.0)	11 (27.5)	13 (32.5)	12 (30.0)	0.607
		3	1 (2.5)	4 (10.0)	13 (32.5)	13 (32.5)	9 (22.5)	
**q2.07**	**Do you think the way the psychologists on the ward interact is adequate?**	2	0 (0)	8 (20.0)	9 (22.5)	10 (25.0)	13 (32.5)	0.840
		3	2 (5.0)	4 (10.0)	8 (20.0)	18 (45.0)	8 (20.0)	
**q2.08**	**Do you think the social support offered on the ward is helpful?**	2	0 (0)	1 (2.5)	14 (35.0)	15 (37.5)	10 (25.0)	0.542
		3	0 (0)	3 (7.5)	10 (25.0)	14 (35.0)	13 (32.5)	
**q2.09**	Do you think the educational support (school in hospital) is satisfactory? *	2	0 (0)	0 (0)	7 (25.0)	14 (50.0)	7 (25.0)	0.084
		3	0 (0)	1 (8.3)	5 (41.7)	5 (41.7)	1 (8.3)	
**q2.10**	**Do you think the games/educational activities are useful?**	2	0 (0)	1 (2.5)	4 (10.0)	13 (32.5)	22 (55.0)	0.537
		3	0 (0)	0 (0)	6 (15.0)	18 (45.0)	16 (40.0)	
**q2.11**	**Do you think the relationships on the ward (between parents) have been helpful?**	2	0 (0)	1 (2.5)	11 (27.5)	15 (37.5)	13 (32.5)	<0.001 ***
		3	1 (2.5)	14 (35.0)	10 (25.0)	10 (25.0)	5 (12.5)	
**q2.12**	**Do you consider the organization and rules of the department to be adequate?**	2	0 (0)	1 (2.5)	6 (15.0)	22 (55.0)	11 (27.5)	0.344
		3	0 (0)	1 (2.5)	9 (22.5)	22 (55.0)	8 (20.0)	
**q2.13**	**Do you think the organization of the outpatient clinic (time slots, waiting room, time available for visits) is adequate?**	2	0 (0)	4 (10.0)	14 (35.0)	14 (35.0)	8 (20.0)	0.458
		3	1 (2.5)	1 (2.5)	14 (35.0)	17 (42.5)	7 (17.5)	
**q2.14**	**Do you think the staff have too high a workload?**	2	0 (0)	2 (5.0)	16 (40.0)	19 (47.5)	3 (7.5)	0.010 *
		3	0 (0)	3 (7.5)	29 (72.5)	7 (17.5)	1 (2.5)	
**q2.15**	**Do you think the staff have too high an emotional load?**	2	1 (2.5)	2 (5.0)	16 (40.0)	15 (37.5)	6 (15.0)	0.523
		3	4 (10.0)	2 (5.0)	17 (42.5)	12 (30.0)	5 (12.5)	
**q2.16**	**Do you think your child’s illness and treatment have had a significant impact on your family’s economic conditions?**	2	6 (15.0)	14 (35.0)	11 (27.5)	7 (17.5)	2 (5.0)	0.957
		3	3 (7.5)	16 (40.0)	15 (37.5)	4 (10.0)	2 (5.0)	
**q2.17**	**Do you feel your child’s illness and treatment have had a significant impact on your family relationships?**	2	1 (2.5)	4 (10.0)	8 (20.0)	15 (37.5)	12 (30.0)	0.253
		3	4 (10.0)	8 (20.0)	9 (22.5)	9 (22.5)	10 (25.0)	
**q2.18**	**Do you think the role of family (grandparents and uncles) and social (friends) emotional resources has been crucial to coping with your child’s disease and treatment?**	2	2 (5.0)	3 (7.5)	10 (25.0)	6 (15.0)	19 (47.5)	0.431
		3	4 (10.0)	4 (10.0)	4 (10.0)	15 (37.5)	13 (32.5)	

* This question has 12 items missing from the 2-COVID-19 dataset and 28 items missing from the 3-COVID-19 dataset. POM: Proportional Odds Model, Wald *p*-value test. *** statistically significant.

## Data Availability

Not applicable.

## Supplementary Materials

The following supporting information can be downloaded at: https://www.mdpi.com/article/10.3390/children9040554/s1, Table S1: Parents’ and patients’ characteristics; Table S2: Details of the p-values obtained with Fisher’s exact test (F) and the Wilcoxon–Mann–Whitney test. (WMW). Table S3. Details of the p-values obtained with Fisher’s exact test (F) and the Wilcoxon-Mann-Whitney test.(WMW).
