# Supplementary material for: Managing Care during the COVID-19 Pandemic: The Point of View and Fears of Pediatric Cancer Patients’ Families
Source: Children (Basel). 2022 Apr 13;9(4):554. doi: 10.3390/children9040554 (PMC9025749; doi:10.3390/children9040554)
Supplement: Supplementary file 1 [file children-09-00554-s001.zip › children-1614979-supplementary.pdf]

Table S1. Parents' and patients' characteristics.

	1	2	3
**Parents' age**			
Median (first and third quartile)	47.5 (39.0 - 51.2)	50.5 (42.0 - 54.2)	43.5 (40.0 - 48.0)
**Gender**			
Female n(%)	24 (60.0)	29 (72.5)	33 (82.5)
Male n(%)	16 (40.0)	11 (27.5)	7 (17.5)
**Region**			
Lombardia n(%)	31 (77.5)	33 (82.5)	30 (75.0)
Other n(%)	9 (22.5)	7 (17.5)	10 (25.0)
**Educational qualifications***			
Secondary-school license n(%)	9 (23.1)	5 (12.5)	7 (17.5)
High-school diploma n(%)	23 (59.0)	21 (52.5)	20 (50.0)
University degree n(%)	7 (17.9)	14 (35.0)	13 (32.5)
**Occupation***			
Lower status employees n(%)	6 (15.4)	3 (7.5)	6 (15.0)
Managers n(%)	1 (2.6)	3 (7.5)	4 (10.0)
Clerks and skilled service employees n(%)	8 (20.5)	17 (42.5)	13 (32.5)
Professionals n(%)	11 (28.2)	11 (27.5)	10 (25.0)
Technicians and associated professionals n(%)	2 (5.1)	2 (5.0)	0
Retired n(%)	1 (2.6)	1 (2.5)	0
Unemployed n(%)	10 (25.6)	3 (7.5)	7 (17.5)
**Patients' age**			
Median (first and third quartile)	12.5 (10.0 - 16.2)	13.0 (4.8 - 17.0)	7.0 (2.8 - 16.0)
**Type of tumor**			
Soft tissue and bone sarcoma n(%)	21 (52.5)	18 (45.0)	15 (37.5)
Carcinoma and melanoma n(%)	1 (2.5)	1 (2.5)	3 (7.5)
CNS tumor n(%)	6 (15.0)	7 (17.5)	8 (20.0)
Lymphoma n(%)	6 (15.0)	5 (12.5)	5 (12.5)
Wilms' tumor and neuroblastoma n(%)	5 (12.5)	7 (17.5)	5 (12.5)
Others n(%)	1 (2.5)	2 (5.0)	4 (10.0)

Table S2. Questionnaire 1: questions and answers.

Item	Not at all n(%)	Slightly n(%)	Moderately/Fairly	Very much n(%)	Extremely n(%)

		n(%)				
**q1.01**	**Are you afraid your child might become infected with COVID-19?**	2 (5.0)	4 (10.0)	21 (52.5)	5 (12.5)	8 (20.0)
**q1.02**	**Are you afraid your child might have serious complications of a COVID-19 infection?**	2 (5.0)	5 (12.5)	23 (57.5)	3 (7.5)	7 (17.5)
**q1.03**	**Are you afraid of becoming infected with COVID-19 yourself?**	3 (7.5)	10 (25.0)	20 (50.0)	2 (5.0)	5 (12.5)
**q1.04**	**Are you afraid of developing serious complications?**	3 (7.5)	11 (27.5)	14 (35.0)	8 (20.0)	4 (10.0)
**q1.05**	**Have you experienced the threat of COVID-19 with anguish?**	4 (10.0)	5 (12.5)	16 (40.0)	11 (27.5)	4 (10.0)
**q1.06**	**Do you think that the changes imposed on society (i.e. closure of shops, gatherings forbidden, stay-home rules except for demonstrable reasons of necessity) have had a strong impact on your daily life?**	4 (10.0)	9 (22.5)	16 (40.0)	6 (15.0)	5 (12.5)
**q1.07**	**Compared to the time before the pandemic, did you experience difficulties or worries about your economic situation being aggravated by having a sick child?**	13 (32.5)	10 (25.0)	11 (27.5)	4 (10.0)	2 (5.0)
**q1.08**	**Do you think there have been family conflicts and communication difficulties in connection with the pandemic?**	20 (50.0)	13 (32.5)	4 (10.0)	3 (7.5)	0 (0)
**q1.09**	**Do you think being unable to physically attend school (all grades) was a problem for your child?**	13 (32.5)	6 (15.0)	12 (30.0)	5 (12.5)	4 (10.0)
**q1.10**	**… and for your family?**	12 (30.0)	12 (30.0)	9 (22.5)	3 (7.5)	4 (10.0)
**q1.11**	**Do you think the suspension of games, sports and social activities outside the hospital was a problem for your child?**	7 (17.5)	8 (20.0)	18 (45.0)	5 (12.5)	2 (5.0)
**q1.12**	**… and for your family?**	5 (12.5)	13 (32.5)	14 (35.0)	5 (12.5)	3 (7.5)
**q1.13**	**Do you think the lack of family ties due to the pandemic (isolation from grandparents, less contact with social networks) has been a problem for your child and for your family in general?**	2 (5.0)	7 (17.5)	17 (42.5)	5 (12.5)	9 (22.5)
**q1.14**	**Do you think the way the pediatric outpatient clinic coped with the emergency (telephone calls to provide updates on a patient's state of health, reserved entrances, body temperature screening at entrance, distribution of hand sanitizer, distribution of face masks) was inadequate, given the situation?**	11 (27.5)	23 (57.5)	6 (15.0)	0 (0)	0 (0)
**q1.15**	**Do you think the changes made to the procedures for managing admissions and outpatient visits (telephone screening,**	11 (27.5)	19 (47.5)	9 (22.5)	0 (0)	1 (2.5)

	**strict time slots, separation of routes for different patients' needs, "telephone visits") had a negative effect?**					
**q1.16**	**Do you think the strict time slot in the outpatient clinic gave doctors less time to devote to your child?**	10 (25.0)	10 (25.0)	19 (47.5)	0 (0)	1 (2.5)
**q1.17**	**Do you think a hospital stay in a single room is less preferable for you?**	15 (37.5)	14 (35.0)	8 (20.0)	0 (0)	3 (7.5)
**q1.18**	**… and for your child?**	16 (40.0)	11 (27.5)	9 (22.5)	2 (5.0)	2 (5.0)
**q1.19**	**Do you think allowing only one parent to stay in hospital with a child (without the opportunity for parents to change places) was a source of difficulty for your family (i.e. for other children left at home with only one parent)?**	9 (22.5)	7 (17.5)	7 (17.5)	6 (15.0)	11 (27.5)
**q1.20**	**… and for your child (i.e. always staying in hospital with mum, without being able to see dad, or vice versa)?**	8 (20.0)	7 (17.5)	7 (17.5)	10 (25.0)	8 (20.0)
**q1.21**	**Do you think banning visits from family and friends during hospital stays was a problem for your family?**	10 (25.0)	9 (22.5)	9 (22.5)	4 (10.0)	8 (20.0)
**q1.22**	**… and for your child?**	9 (22.5)	9 (22.5)	10 (25.0)	3 (7.5)	9 (22.5)
**q1.23**	**Do you think the reduction of games and educational activities to support community life on the ward was a problem for your family?**	7 (17.9)	10 (25.6)	10 (25.6)	6 (15.4)	6 (15.4)
**q1.24**	**… and for your child?**	6 (15.0)	12 (30.0)	11 (27.5)	6 (15.0)	5 (12.5)
**q1.25**	**Do you think conducting supportive interviews with doctors and psychologists while wearing personal protective equipment (such as masks) and adopting distancing measures was unsatisfactory?**	8 (20.0)	19 (47.5)	12 (30.0)	0 (0)	1 (2.5)
**q1.26**	**Do you think conducting supportive interviews with doctors and psychologists while adopting distancing measures may have created potential opportunities for misunderstandings, undermining the effectiveness of such interventions?**	18 (45.0)	6 (15.0)	7 (17.5)	7 (17.5)	2 (5.0)
**q1.27**	**During the pandemic did you perceive an increase in the level of stress among care operators?**	12 (30.0)	9 (22.5)	15 (37.5)	3 (7.5)	1 (2.5)
**q1.28**	**In your experience during the pandemic, do you feel that you received less attention during your child's cancer treatment?**	34 (85.0)	1 (2.5)	4 (10.0)	1 (2.5)	0 (0)
**q1.29**	**In your experience during the pandemic, do you feel that you received less**	30 (75.0)	5 (12.5)	3 (7.5)	1 (2.5)	1 (2.5)

	attention regarding the psychosocial sphere?					
q1.30	In your experience during the pandemic, do you feel that you received less attention in the area of nursing care?	36 (90.0)	2 (5.0)	2 (5.0)	0 (0)	0 (0)
q1.31	Do you think the COVID-19 pandemic has affected the quality of care your child received?	33 (82.5)	4 (10.0)	0 (0)	2 (5.0)	1 (2.5)
q1.32	Do you feel that concern about the COVID-19 pandemic has outweighed concern about your child's illness?	30 (75.0)	9 (22.5)	1 (2.5)	0 (0)	0 (0)

Table S3. Details of the p-values obtained with Fisher's exact test (F) and the Wilcoxon-Mann-Whitney test.(WMW).

	Item	p-value F	p-value WMW
1	**Do you consider the medical care to have been clinically satisfactory?**	0.232	0.179
2	**Do you think the medical care was satisfactory in terms of communication?**	0.358	0.264
3	**Do you think the time spent by doctors during outpatient visits was adequate?**	0.547	0.773
4	**Do you think the time spent by doctors during inpatient visits was adequate?**	0.830	0.629
5	**Do you think the nursing care was satisfactory?**	0.421	0.199
6	**Do you feel you received the psychological support you needed during your child's treatments?**	0.921	0.412
7	**Do you think the way the psychologists on the ward interact was adequate?**	0.145	0.846
8	**Do you think that the social support offered on the ward is helpful?**	0.564	0.631
9	**Do you think the educational support (school in hospital) is satisfactory? ***	0.233	0.071
10	**Do you think the games / educational activities are useful?**	0.345	0.252
11	**Do you think the relationships on the ward (between parents) have been helpful?**	< 0.001***	< 0.001***
12	**Do you consider the organization and rules of the department to be adequate?**	0.776	0.358
13	**Do you think the organization of the outpatient clinic (time slots, waiting room, time available for visits) is adequate?**	0.612	0.747
14	**Do you think the staff have too high a workload?**	0.008**	0.003**
15	**Do you think the staff have too high an emotional load?**	0.716	0.292
16	**Do you think your child's illness and treatment have had a significant impact on your family's economic conditions?**	0.642	0.873
17	**Do you feel your child's illness and treatment have had a significant impact on your family relationships?**	0.330	0.097
18	**Do you think the role of family (grandparents and uncles) and social (friends) emotional resources has been crucial to coping with your child's disease and treatment?**	0.078	0.449
